# Liquid biomarkers in melanoma: detection and discovery

**DOI:** 10.1186/s12943-018-0757-5

**Published:** 2018-01-17

**Authors:** Su Yin Lim, Jenny H. Lee, Russell J. Diefenbach, Richard F. Kefford, Helen Rizos

**Affiliations:** 1 Faculty of Medicine and Health Sciences, Macquarie University, Sydney, NSW Australia; 2 Melanoma Institute Australia, Sydney, NSW Australia; 3 Department of Medical Oncology, Crown Princess Mary Cancer Centre, Westmead and Blacktown Hospitals, Sydney, NSW Australia; 4 Department of Biomedical Sciences, Faculty of Medicine and Health Sciences, Macquarie University, 2 Technology Place, Sydney, NSW 2109 Australia

**Keywords:** Melanoma, Liquid biopsy, Biomarkers, Immunotherapy, Targeted therapy

## Abstract

A vast array of tumor-derived genetic, proteomic and cellular components are constantly released into the circulation of cancer patients. These molecules including circulating tumor DNA and RNA, proteins, tumor and immune cells are emerging as convenient and accurate liquid biomarkers of cancer. Circulating cancer biomarkers provide invaluable information on cancer detection and diagnosis, prognosticate patient outcomes, and predict treatment response. In this era of effective molecular targeted treatments and immunotherapies, there is now an urgent need to implement use of these circulating biomarkers in the clinic to facilitate personalized therapy. In this review, we present recent findings in circulating melanoma biomarkers, examine the challenges and promise of evolving technologies used for liquid biomarker discovery, and discuss future directions and perspectives in melanoma biomarker research.

## Background

The overall survival of patients with Stage III and IV melanoma has improved dramatically in the last ten years with the introduction of immunotherapies and mitogen activated protein kinase (MAPK) targeted treatments [1–3]. These therapies produce durable responses in 20% of melanoma patients, with survival extended up to 10 years in a proportion of patients treated with the immune checkpoint inhibitor ipilimumab [4, 5]. Both MAPK and immune checkpoint inhibitor therapies have significant limitations, however. Targeted therapies are limited by the emergence of drug resistance in the majority of patients within 12 months of therapy initiation [5], single-agent immunotherapies benefit only 10–40% of patients [6, 7], and the combination of immune checkpoint inhibitors produces significant toxicities [7, 8] (Table 1). In the case of immunotherapies, the activity of these agents are further complicated by pseudo-progression, heterogeneous response and delayed regression [9, 10].

**Table 1:** Systemic melanoma therapies: Phase III clinical trial outcomes

Therapy	ORR	Median PFS (months); % survival (year)	Median OS (months); % survival (year)	Grade 3/4 toxicity	Biomarkers examined	Reference
Molecular therapies						
Vemurafenib^1^ (*n* = 337)	48%	6.9; 14% (1.5 years)	13.6; 39% (1.5 years)	73%	BRAF V600 mutation and LDH [131]	[132, 133]
Dabrafenib^1^ (*n* = 187)	50%	5.1; 12% (3 years)	20; 45% (2 years)	53%^¥^	BRAF V600 mutation and LDH [134]	[135, 136]
Trametinib^2^ (*n* = 214)	22%	4.8; NR	NR; 81% (6 months)	NR	BRAF V600 mutation and LDH [137]	[138]
Dabrafenib + trametinib (*n* = 352)	64%	12.1; 30% (2 years), 24% (3 years)	25.6; 73% (1 year), 52% (2 years), 44% (3 years)	52%	BRAF V600 mutation and LDH [139]	[139, 140]
Vemurafenib + cobimetinib^2^(*n* = 247)	70%	12.3; NR	22.3; 75% (1 year), 48% (2 years)	60%	Ki67, p56, MAPK, PI3K pathways, cell proliferation, CD8 T cells [141]	[141]
Immunotherapies						
gp100^3^ (*n* = 136)*	1.5%	2.8; 48.5% (12 weeks)	6.4; 25.3% (1 year), 13.7% (2 years)	11.4%	LDH [3]	[3]
Ipilimumab^4^ (*n* = 278)	13%	2.8; 14% (2 years)	16.0; 43% (2 years)	20%	LDH, peripheral blood absolute lymphocyte count [142]	[6, 143]
gp100 + ipilimumab (*n* = 403)*	5.7%	2.8; 49.1% (12 weeks)	10; 44% (1 year), 21.6% (2 years)	17.4%	LDH [3]	[3]
Nivolumab^5^ (*n* = 210)	40%	5.1; 44% (1 year)	Not reached; 73% (1 year)	11.7%	Tumor cell PD-L1 expression, peripheral blood absolute lymphocyte count [142]	[144]
Pembrolizumab^5^ (*n* = 277)	36%	4.1; 28% (2 years)	Not reached; 55% (2 years)	17%	LDH, blood count parameters [129]	[6, 143]
Ipilimumab + nivolumab (*n* = 314)	57.6%	11.5; 49% (1 year), 39% (3 years)	Not reached; 64% (2 years), 58% (3 years)	55%	Tumor cell PD-L1 expression, peripheral blood absolute lymphocyte count [142]	[7, 145]
T-VEC^6^ (*n* = 295)	26.4%**	NR	23.3; 50% (2 years)	36%	None	[146]

ORR, objective response rate; NR, Not reported; PFS, progression free survival; OS, overall survival. Grade 3/4 toxicity as defined by the American National Institute of Health and National Cancer Institute’s Common Terminology Criteria for Adverse Events version 4.0 (CTCAE). *PFS only available at 12 weeks; ** Durable response rate was used and not standard RECIST criteria; ^¥^Grade ≥ 2 only, as grade 3/4 not reported

^1^Dabrafenib and vemurafenib are selective BRAFV600 inhibitors. ^2^Trametinib and cobimetinib are inhibitors of MEK1/2. ^3^gp100 is a human melanoma peptide vaccine. ^4^Ipilimumab is an antibody targeting the CTLA-4 receptor. ^5^Nivolumab and pembrolizumab are antibodies targeting the PD-1 receptor. ^6^T–VEC (talimogene laherparepvec) is a genetically engineered oncolytic virus. LDH denotes lactate dehydrogenase

In this era of multiple effective therapies, designing the optimal treatment strategy for each cancer patient requires the development of sophisticated diagnostic, prognostic and predictive biomarkers that are sensitive and specific for cancer detection, patient outcomes and treatment response. The ideal biomarker in metastatic melanoma would guide sequencing and identify the optimal timing to introduce the next line of therapy, and differentiate patients who would benefit from treatment beyond progression. The latter is particularly important in the case of pseudo-progression, which can occur both early and late into the treatment. With recent publications outlining the effectiveness of adjuvant targeted and immunotherapies in stage III melanoma [11, 12], a newfound role for biomarkers that identify patients most likely to benefit from adjuvant treatment is emerging. Indeed, several recent clinical trials have now included evaluation of tissue and blood-based biomarkers as secondary endpoints or additional assessment parameters (Table 1).

**Fig. 1:**
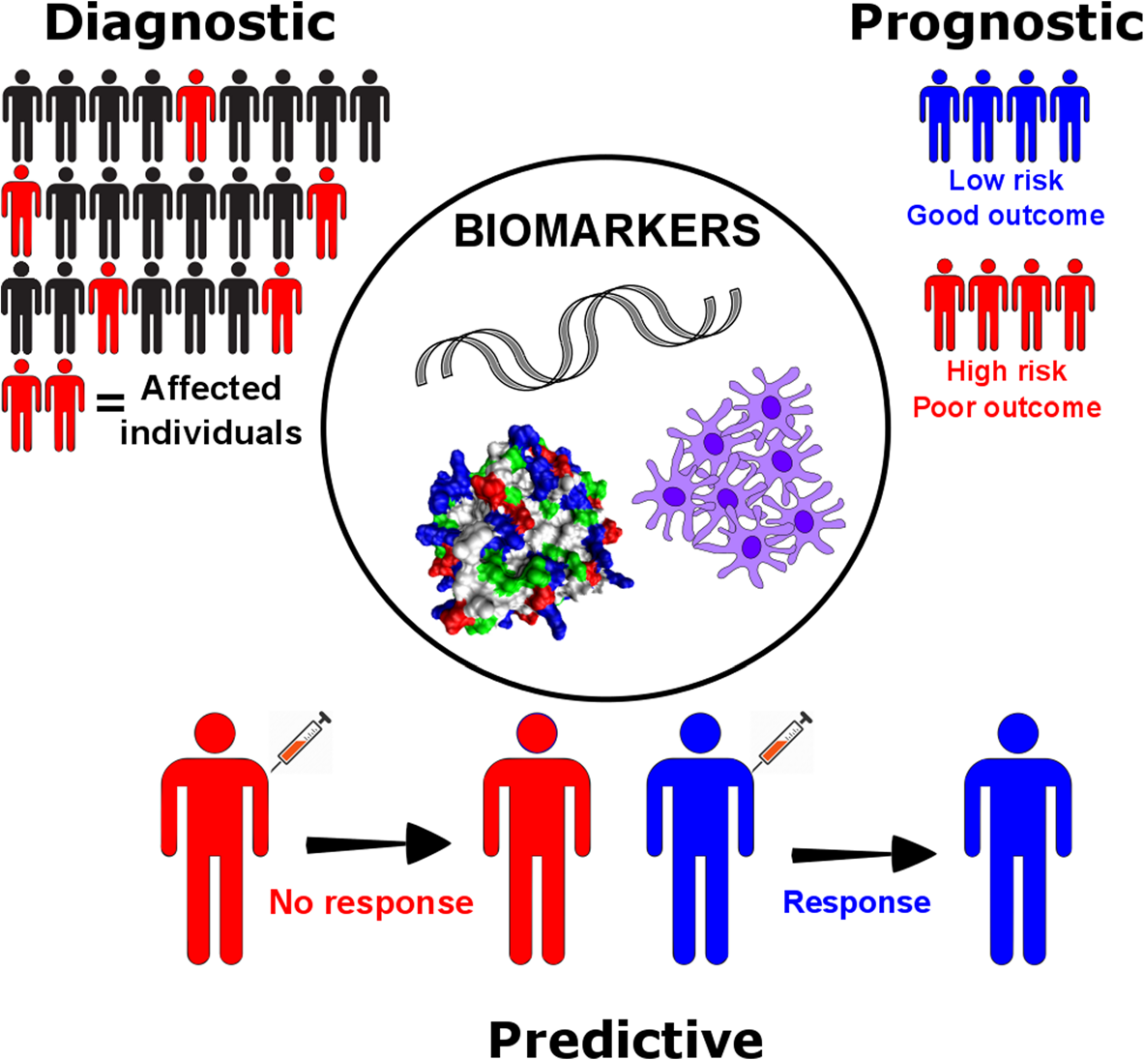
Clinical applications of cancer biomarkers. Genetic, protein and cellular components can serve as diagnostic, prognostic and/or predictive biomarkers of cancer. Diagnostic biomarkers are used to identify and detect presence of cancer in individuals, prognostic biomarkers provide information on disease progression and expected outcomes, and predictive biomarkers forecast the likely benefit of a specific treatment

Cancer biomarkers can be classified as diagnostic, prognostic or predictive (Fig. 1). Diagnostic biomarkers identify and confirm the presence of cancer to facilitate early detection, prognostic markers forecast the probable course and likely outcomes of a disease regardless of treatment, and predictive biomarkers evaluate the likelihood of benefit from a specific treatment [13]. The presence or absence of a prognostic marker can be used clinically to triage patients into optimal treatment strategies, and predictive markers facilitate personalized therapy [14, 15].

Circulating biomarkers identified in biological liquid samples, termed liquid biopsies, are particularly valuable as they can be sampled repeatedly in real time and are non-invasive [16]. Here, we describe progress in melanoma liquid biomarker discovery, discuss the promise and limitations of emerging technologies and highlight future directions and perspectives in cancer biomarker research.

## Clinical biomarkers in melanoma: Current status

There are many prognostic and predictive biomarkers used clinically in melanoma, and these form the updated version of the 8th edition American Joint Committee on Cancer (AJCC) melanoma staging system [17]. This staging system relies on the histological characteristics of melanoma, including tumor thickness, ulceration and mitotic rate (Table 2). The only circulating protein biomarker with significant prognostic value in the AJCC staging system is lactate dehydrogenase (LDH) [17]. Elevated LDH correlates with poor survival in stage IV melanoma [18] and is an independent predictor of poor outcome in patients treated with combination dabrafenib and trametinib [19]. Moreover, a significant reduction in LDH (i.e. mean LDH decrease of 27.3% from baseline) is associated with response to immunotherapy on first CT scan [20]. Several other circulating proteins have shown diagnostic and prognostic value for melanoma, including S100B, C reactive protein (CRP) and melanoma-inhibiting activity (MIA) protein (reviewed in [21]) but all have limitations in routine clinical use.

**Table 2:** Clinical biomarkers for the prognosis and prediction of melanoma

	Characteristics	Associated with worse outcomes	Reference
Prognostic biomarkers			
Primary melanoma	Thickness	Thick melanomas	[147]
	Ulceration	Present	[147]
	Histology	Nodular and acral subtype	[148]
	Mitotic rate	Presence of mitosis	[149]
	Age	> 60	[147, 149]
	Site	Trunk, head and neck	[147, 149]
	Mutation status	BRAF or NRAS positive	[150]
Stage III melanoma	Lymph node stage (AJCC)	IIIC	[151]
	Nodal status	Increased number of positive lymph nodes	[147]
	Tumor burden	Macroscopic disease	[147]
	Ulceration on primary melanoma	Present	[147]
	Extracapsular extension	Present	[151]
Metastatic melanoma	Distant metastatic site	Visceral metastasis	[147, 152]
	Number of visceral metastasis	≥ 2	[152, 153]
	LDH	Above upper limit of normal	[152, 153]
	Serum albumin	< 3.5 g/deciliter	[153]
	ECOG performance status	≥ 1	[152]
	Hematological parameters	Abnormal platelets	[152]
Predictive biomarkers			
MAPK therapy	BRAF Status	No BRAF V600 mutation	[131]
	LDH	Above upper limit of normal	[19]
	ECOG performance status	≥ 1	[19]
	Number of organ sites containing metastases	≥ 3	[19]
	Sites of disease	Visceral only	[19]
	Baseline disease stage	IVM1c	[19]
	Sum of lesion diameter	≥ median (58 mm)	[19]
Immunotherapy	LDH	Above upper limit of normal	[154]
	Baseline tumor size	≥ median (102 mm)	[154]
	Stage	IVM1a or IVM1c	[154]

## Liquid biomarkers in melanoma

**Fig. 2:**
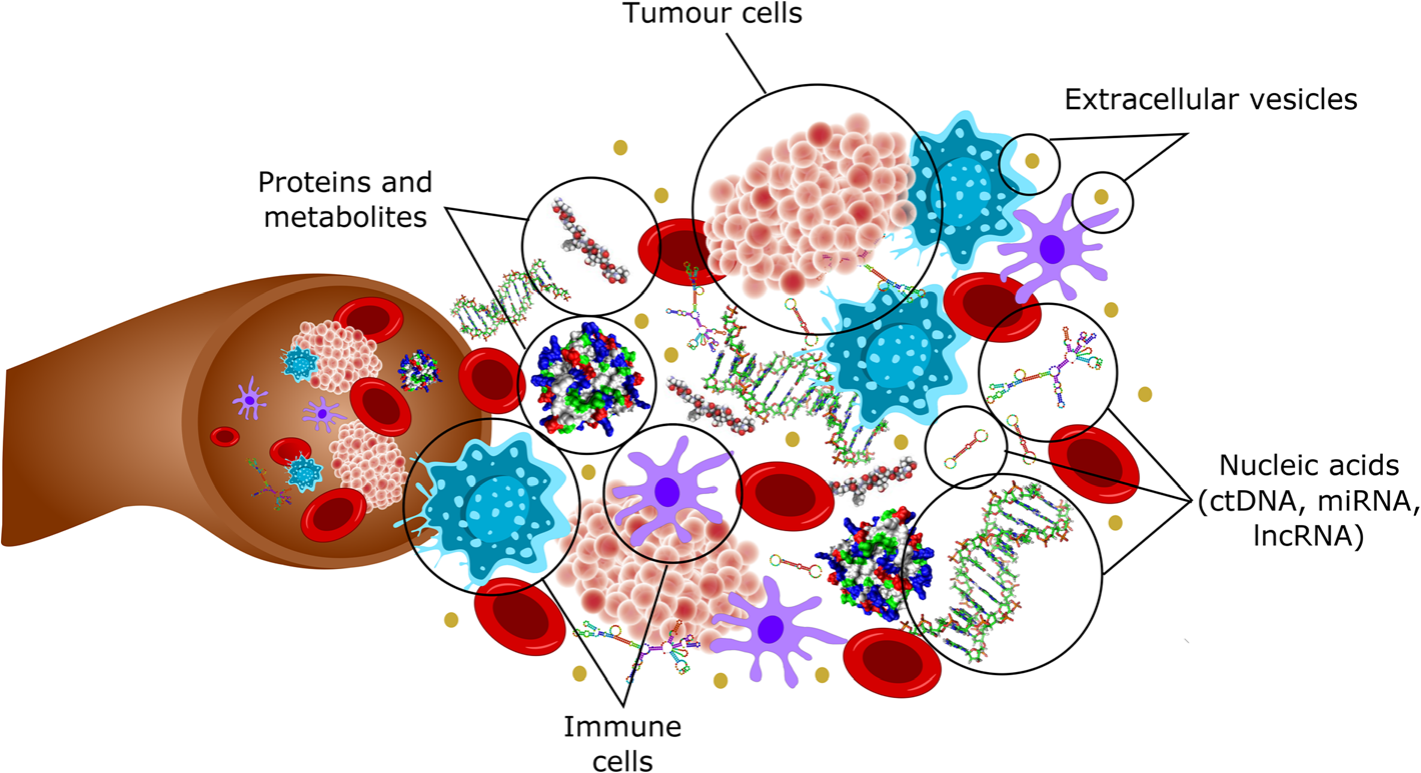
Circulating biomarkers. Tumour and immune cells, proteins, nucleic acids and extracellular vesicles (which include exosomes) can be detected in circulation and may serve as potential cancer biomarkers. ctDNA, circulating tumour DNA; miRNA, micro RNA; lncRNA, long non-coding RNA

Tumor cells, tumor-derived metabolites, proteins, nucleic acids and vesicles are constantly shed into the circulation and these circulating components can provide valuable diagnostic, prognostic and predictive information (Fig. 2). Liquid biopsies can capture circulating components and offer several advantages to tissue based profiling; they are minimally invasive, can profile clonally divergent, distant metastases without sampling bias, and allow for routine longitudinal tracking of patient response to therapy [16]. Tumors within a single patient are genetically and phenotypically diverse, and liquid biopsies, which can contain molecules derived from multiple metastases [22, 23], may provide a more comprehensive profile of a patient’s tumor burden [22]. Moreover, clonal evolution and selection on systemic cancer therapies occurs rapidly, and longitudinal measurements with liquid biopsies can monitor disease progression, subclone evolution and patient response [24].

Although many circulating cancer biomarkers have been identified in pre-clinical models and clinical samples, few have been validated or FDA-approved for clinical use. A critical review in 2005 showed a general decline in FDA-approved plasma protein biomarkers, despite an increase in biomarker-related publications during this period. For instance, of the 2000 publications on cancer biomarkers in 1994, two plasma protein biomarkers received FDA approval and only one biomarker was approved by the FDA in 2000, despite over 3000 biomarker publications [14]. The incongruity between biomarker identification and clinical implementation reflects the significant intra- and inter-patient variation (i.e. fluctuations in plasma protein levels between different patients over time), the inadequacies in current technologies (i.e. limitation in specificity and sensitivity), and differences in processing and analytical methods (i.e. lack of consistency in blood sampling, storage and processing).

In the following sections, we discuss commonly used proteomic, molecular and cellular profiling approaches, the progress and limitations of these emerging technologies, and their contributions to melanoma biomarker discovery (Table 3).

**Table 3:** Advantages and disadvantages of current technologies in biomarker discovery

Biomarker	Detection Technology	Advantages	Disadvantages
Proteins and peptides	Mass spectrometry	High specificity, accurate identification of proteins	Requires significant optimization, time-consuming, limited dynamic range of detection, affected by abundant proteins
	Affinity-based multiplex assays	High throughput, allows absolute quantification, requires small sample amounts, does not require depletion of abundant proteins	Detection limited to selected protein targets, potential cross reactivity of antibodies or aptamers may contribute to false positives
ctDNA	Digital PCR	Cost effective, high accuracy and reproducibility	Lacks standardization and is limited to 1–2 mutations per test
	BEAMing	High sensitivity, accuracy, and reproducibility	Lacks standardization and is limited to a single mutation per test
	Next generation sequencing	Allows large-scale coverage	Costly and complex, low sensitivity
miRNA and lncRNA	Quantitative PCR	Widely used, straightforward, and cost effective	Requires a standard curve and specificity is dependent on primer design
Exosomes	ExoScreen	High throughput, requires small sample amounts, and eliminates complicated isolation steps	Lacks normalization and standardization
Circulating tumor cells	Cell Search	Highly specific and robust, and has minimal variability	Requires known cell surface marker (i.e. EpCAM) to capture cells
	Slated spiral microfluidics	Fast processing time and cost effective	Requires large sample volume
Circulating immune cells	Flow cytometry	High throughput, able to screen multiple markers simultaneously	Limited number of markers due to spectral overlap
	Mass cytometry	High throughput, able to screen multiple markers simultaneously	Requires significant expertise, slow acquisition rate and requires more stringent sample preparation

### Proteomic profiling for liquid biomarker discovery in melanoma

Proteins are easily recovered from blood plasma and serum, but the high abundance of a few proteins, including albumin (55% of all plasma proteins [25]), the broad range of protein sizes (50–20,000 kDa), and the 9–10 orders of magnitude reported for plasma protein concentrations complicate the detection of low-abundant protein biomarkers [26]. Moreover, protein levels can vary significantly between serum and plasma, and this can be influenced by storage conditions (i.e storage time and temperature), the method of blood fractionation and the properties of the specific protein/s being analyzed [27, 28]. Thus, there is significant discrepancy in the literature regarding protein levels in plasma and serum [29–31], although plasma has shown better reproducibility in protein measurement [31]. Despite the discrepancies, there are now several mature technologies available for plasma and serum protein identification and quantification, including mass spectrometry proteome profiling and affinity-based methods.

#### Mass spectrometry proteome profiling

Mass spectrometry (MS) is based on the fractionation of proteolytic peptides by liquid chromatography and subsequent quantitation and characterization of each fraction. MS-based technologies require significant optimization, expertise and are time consuming (Table 3). They often have limited sensitivity and dynamic range, detect proteins at or above the microgram level, and over a dynamic range of only six orders of magnitude [32]. MS-based techniques are also affected by abundant proteins and many strategies have been employed to deplete these abundant proteins, including ultrafiltration, solid phase and organic solvent extraction, and serum or plasma fractionation. Each of these depletion strategies has disadvantages (reviewed in [32]) and there remain reservations about depleting high abundance proteins due to the removal of non-targeted proteins [33]. Instead of depletion, some studies have also attempted to enrich specific target proteins by affinity capture but this limits the high-throughput biomarker discovery capabilities of MS [33].

There have been many refinements in MS-based technologies to improve throughput and quantitation, including multiple reaction monitoring (MRM) and sequential window acquisition of all theoretical mass spectra (SWATH-MS). Highly multiplexed MRM platforms are capable of quantitating proteins and have been used to identify potential biomarkers in bladder cancer [34]. SWATH-MS also offers high throughput quantification of protein biomarkers using fragment-ion intensity-based quantification [35]. MRM and SWATH-MS detect a defined set of protein targets within a specific peptide library [35], and these are evolving into highly specific, quantitative methods, and are particularly attractive when specific antibodies are not available [36]. Discrepancies in protein identification and quantitation occur due to bias introduced during bioinformatics analysis of high abundance molecules and peak identity [37], and as a result, there have been few independent validation studies. For example, although analysis of serum protein profiles using MS in melanoma patients with stage I to IV melanoma accurately predicted disease stage [38] and disease recurrence [39, 40], these initial findings have not been validated in larger independent cohorts. Thus far, there have not been any melanoma biomarkers identified by MS-based techniques that have extended into clinical applications.

#### Affinity-based proteomic assays

Affinity-based proteomic assays capture target proteins and utilize a secondary detection method to generate a quantifiable signal proportional to the quantity of protein present in samples. Traditional singleplex assays such as the enzyme-linked immunosorbent assay (ELISA) detect a specific protein, but more contemporary multiplex methods simultaneously measure many target proteins over a wide dynamic range without the need for the depletion of abundant proteins. These assays are dependent on highly specific antibodies or modified aptamers. For instance, the Mesoscale Discovery Technology Platform (MSD) and the Q-Plex array (Quansys Biosciences) have capture antibodies immobilized on a solid phase support, while the Luminex (ThermoFisher), Cytometric Bead Arrays (BD Biosciences) and Bio-PlexPro (BioRad) assays utilize antibodies conjugated to fluorescently-activated microbeads to allow identification and detection using a flow cytometry-based method [41, 42]. The SOMAscan technology utilizes modified aptamers, or SOMAmers, which are short strands of DNA that recognize specific target epitopes [43, 44].

Affinity-based protein profiling assays depend on antibodies or aptamers that recognize specific epitopes, without cross-reactivity to other proteins. Although capture antibodies or aptamers are analyzed for cross reactivity, they are often tested against a restricted panel of antigens. Method testing and validation of these multiplex immunoassays are critical but few studies have examined performance of these assays in detail [41]. Indeed, we recently compared a bead-based and an aptamer-based affinity assay and found poor correlation in relative plasma protein quantification between the two assays [45]. This highlights the discrepancies introduced when using different assays, which will limit the comparison and validation of potential biomarkers in independent studies. In fact, although there have been several promising circulating biomarkers identified using affinity-based profiling approaches, these have not been validated. For instance, high serum levels of VEGF (more than 43 pg/ml) at baseline was associated with decreased overall survival in stage IV metastatic melanoma patients treated with ipilimumab [46], and serum CXCL8 levels correlated with melanoma proliferation and survival in 24 BRAF-mutant melanoma patients treated with MAPK inhibitors [47]. However, whether VEGF or CXCL8 can be considered robust biomarkers to be applied in the clinic has not been further explored.

### Molecular profiling for liquid biomarker discovery in melanoma

Genetic and signaling changes that drive melanoma development and progression can be identified through molecular profiling. These changes can be detected in the circulation in the form of circulating free DNA (cfDNA), including circulating tumor DNA (ctDNA), microRNA (miRNA) and long non-coding RNA (lncRNA) that are shed into the blood stream by cancer cells. Detection sensitivity of cfDNA is a persistent problem, however, and a number of studies have sought to optimize the yield and stability of cfDNA by comparing a range of tubes during blood collection [48, 49], and a range of commercial cfDNA purification kits [50, 51]. The hope from such studies is to standardize practices in the field with the aim to enhance both sensitivity and consistency.

Commercial cfDNA purification kits typically employ a spin column-based or magnetic bead-based approach. Spin columns are more time consuming and costly, but appear to be the more consistent with higher yields than the magnetic-based systems [48, 49]. Both approaches have the capacity to process large volumes of plasma, an important consideration for maximizing sensitivity, and can be partially or fully automated, which is attractive for high throughput, especially in a diagnostic setting. However, there is currently no standard best practice for cfDNA extraction.

#### Circulating tumor DNA (ctDNA)

ctDNA is highly fragmented single or double stranded DNA shed by tumor cells into the circulation [52]. ctDNA has a size distribution of 130–170 bp, which is equivalent to the size of nuclease-cleaved nucleosomes, and suggestive that cell apoptosis is the principal source of ctDNA. Nevertheless, the precise mechanism of ctDNA release remains to be determined and may potentially include tumor cell necrosis, secretion from metabolically active tumor cells, or phagocytosis of necrotic tumor cells by macrophages [53]. ctDNA has a short half-life, ranging from 16 min to 13 h [54, 55], due to its rapid clearance from circulation via the kidneys, liver and spleen [56].

The utility of ctDNA in identifying heterogeneous resistance mechanisms to EGFR targeted therapy has been well outlined in non-small cell lung cancer [57], with the National Comprehensive Cancer Network guidelines recommending the use of liquid biopsy as an alternative to tissue biopsy for initial T790M mutation testing [58]. However, the role of identifying mutations through ctDNA guiding treatment decisions has not been established, with only case reports available [59].

Levels of ctDNA in cancer patients are associated with disease volume and can be influenced by tumor location, vascularity and cellular turnover [60, 61], and ctDNA is often undetectable in the majority of early stage melanoma patients [62]. However, in late stage melanomas, longitudinal assessment of ctDNA levels, including BRAF and NRAS mutations in ctDNA, in melanoma patients receiving immunotherapy was predictive of response [63]. A favorable ctDNA profile (i.e. undetectable ctDNA either at baseline or during treatment) was associated with a better objective response, progression free and overall survival compared to patients with an unfavorable ctDNA profile (i.e. detectable ctDNA at baseline which remained detectable during therapy) [63]. Similarly, baseline levels of ctDNA were lower in melanoma patients with better outcomes on targeted therapy (reviewed in [64, 65]). In metastatic uveal melanoma, ctDNA was associated with tumor burden and overall survival [66].

In addition to ctDNA quantitation, epigenetic changes in ctDNA such as methylation can also be detected and analyzed. Epigenetic modifications of ctDNA, especially the evaluation of methylation signatures [67], is a promising avenue for biomarker discovery [68, 69]. The stability of CpG island methylation, and the high rate of occurrence early in cancer make methylation analysis of ctDNA a reliable and sensitive biomarker target [67]. Analysis of methylated ctDNA requires bisulfite conversion, which involves the deamination of unmethylated cytosines to uracil to allow discrimination of unmethylated from methylated cytosines. This involves PCR-based amplification using discriminating methylation specific primers for individual methylation sites, or non-discriminating primers coupled with sequencing for a more global gene analysis [70]. Other common methylation analysis techniques are based on the use of methylation sensitive restriction enzymes [70]. The analysis of methylated ctDNA using methylation-specific PCR in metastatic melanoma has yielded promising associations, namely in hypermethylation of the promoter region of Ras association domain family protein 1 (RASSF1A) significantly correlating with overall survival [71] and hypermethylation of estrogen receptor α predicting progression-free and overall survival [72].

Many different platforms have been used to detect ctDNA including quantitative PCR (qPCR), digital droplet PCR (ddPCR) and next generation sequencing [73] (reviewed in [61, 64], Table 3). Several technologies have also been developed to improve detection rate, including crosslinking ctDNA to magnetic beads (BEAMing), enrichment for mutant alleles (i.e. SCODA, synchronous coefficient of drag alteration; COLD-PCR, co-amplification at lower denaturation temperature PCR) and targeted hybrid selection and capture (i.e. CAPP-Seq, cancer personalized profiling by deep sequencing) (reviewed in [74]).

There are a number of challenges in ctDNA detection and analysis. The proportion of ctDNA is low compared to total background cfDNA, and it is imperative that white blood cell lysis, which increases the cfDNA fraction, is avoided during pre-analytical steps such as blood collection, processing and storage. Plasma is the preferred source for ctDNA compared to serum due to greater cell lysis that occurs during the clotting process [75]. However, there is also a lack of consistency in blood processing and plasma preparation, which may affect ctDNA quantitation, especially since ctDNA has a short half-life and there is a time-dependent increase in cfDNA in blood collection tubes [76]. Factors such as time from blood collection to plasma separation, and the temperature for storage and transportation of collected blood are crucial in minimizing cell lysis and maintaining a stable cfDNA pool. Several blood collection tubes have been manufactured from companies such as Streck, Roche, Qiagen and CellSearch which minimize cell lysis and stabilize the total cfDNA pool by the inclusion of various additives/preservatives.

Currently, detection of BRAF and NRAS mutations in ctDNA has shown significant value in predicting treatment response and outcome in melanoma [63, 65, 77] and the recent inclusion of ctDNA analysis in clinical trials [64] further highlights its imminent implementation in clinical practice.

#### MicroRNAs (miRNAs) and long noncoding RNAs (lncRNAs)

MicroRNAs are short (20–200 nucleotides) noncoding RNA molecules that regulate gene transcription processes to affect cell proliferation, apoptosis, differentiation and survival. Long noncoding RNAs (lncRNAs), spanning more than 200 nucleotides, also have direct roles in transcriptional, post-transcriptional and epigenetic gene expression modulation [78]. Both miRNAs and lncRNAs are secreted by cells into the circulation, and unlike ctDNA, they are relatively stable as they are predominantly secreted in vesicles, or in complex with other proteins such as high density lipoprotein and RNA-binding proteins [79–82]. miRNAs and lncRNAs have been implicated in regulation of tumor development, progression and metastasis, and as such, have been proposed as potential cancer biomarkers (reviewed in [78, 83]).

Detection of miRNAs requires selective and sensitive amplification methods including isothermal exponential amplification and rolling cycle amplification, capillary electrophoresis-based assays, and use of quantum dots, Raman spectroscopy, gold nanoparticle probes and duplex specific nucleases [84]. Currently, levels of miRNAs in serum or plasma are normalized against housekeeping control miRNAs, such as U6, miR-451 and miR-16, or with spiked-in controls. However, levels of these controls may be deregulated in cancer and spiked in controls may not be practical when dealing with large numbers of biological samples [85]. Unbiased RNA detection methods are also required for lncRNA detection and analysis, and these typically include tiling arrays, where cDNA is hybridized to microarray slides containing overlapping oligonucleotides that cover the complete genome, serial analysis of gene expression (SAGE) and cap analysis of gene expression (CAGE), which involve sequencing of short cDNA sequences [86].

Expression of miRNAs and lncRNAs have shown diagnostic, prognostic and predictive value in melanoma [87]. However, it is important to emphasize that miRNAs and lncRNAs are not tumor specific and it is difficult to attribute whether changes in abundance are due to the cancer or to secondary conditions such as inflammation [88]. Elevated levels of miRNA-221 have been observed in early melanomas compared to healthy controls and melanoma in situ and increasing miRNA-221 levels further correlated with increased stage [89]. Additionally, a panel of five miRNAs (miRNA-150, miRNA-15b, miRNA199a-5p, miRNA-33a and miRNA-424) classified primary melanoma patients into high-risk compared to low-risk of recurrence, and dynamic changes in longitudinal samples reflected tumor burden [90]. Several lncRNAs are also upregulated in melanoma including SPRY4-IT1, BANCR, HOTAIR, UCA1 and MALAT-1 [91]. Levels of UCA1 and MALAT-1 were significantly upregulated in melanomas compared to normal controls, and were significantly higher at later stage (stage III and IV) compared to early stage melanomas (stage I and II) [85]. Overall, these studies implicate miRNAs and lncRNAs as promising prognostic and predictive biomarkers for melanoma but because there have been few studies in this area, and no additional studies have been performed to validate these findings, use of miRNAs and lncRNAs as biomarkers have not been translated into clinical use.

#### Exosomes

Exosomes are small (30–150 nm; equivalent to viruses) membrane bound vesicles, produced by all cells and capable of transporting DNA, RNA and proteins between cells. Tumor cells actively secrete exosomes, and these can deliver tumor-specific cargo (DNA, RNA and protein) to other body sites to modify tumor survival, proliferation and treatment response [92, 93]. Secreted exosomes can be isolated based on their physical properties using ultracentrifugation, size-based methods, precipitation-based assays, immune-affinity capture and microfluidics (reviewed in [94]). Characterization of quality and integrity of isolated exosomes typically include transmission electron microscopy, which captures vesicle morphology and size, coupled with a complementary analysis which measures size distribution and concentration of exosomes (reviewed in [95]).

Exosome levels can be monitored directly in the circulation via cell surface markers such as the tetraspanin proteins, CD63, CD81 and CD9. For example, ExoScreen can detect and quantitate exosome surface proteins with streptavidin-coated donor beads that capture analyte-specific biotinylated antibodies, and acceptor beads conjugated to secondary antibodies that recognize an epitope of the analyte [96]. ExoScreen is superior to immunoblotting detection of exosomes, as it does not require exosome purification or concentration (Table 3). Exosome cargo may also serve as cancer biomarkers, and elevated levels of exosome-derived miRNA-17, miRNA-19a, miRNA-21, miRNA-126 and miRNA-149 were identified in patients with sporadic metastatic melanoma compared to healthy individuals [97]. Further promise in this area is illustrated by the recent report that miRNA-211-5p was induced within exosomes in response to vemurafenib treatment of BRAF-mutant melanoma cells, and as such, may represent a potential biomarker or therapeutic target [98].

#### Cellular profiling in liquid biomarker discovery

Whole blood contains different inflammatory and immune cell subsets such as peripheral blood mononuclear cells (PBMCs), and can also contain circulating tumor cells (CTCs), derived from malignant cells that have detached from primary or metastatic tumor sites and shed into the circulation. The phenotypic and functional analysis of whole blood may identify potential cell-based biomarkers [99].

#### Circulating tumor cells (CTCs)

CTCs represent a small proportion of cells in the circulation, and are detected at a rate of approximately one CTC per million leukocytes [100]. The detection of CTCs is further complicated by their short half-life of 1 to 2.4 h in circulation [101]. CTC counts reflect tumor burden and the presence of CTCs strongly correlated with poor outcome in several cancers [102, 103]. Furthermore, changes in CTC counts during therapy have been associated with treatment response [104, 105].

Currently, there are more than 400 clinical trials incorporating CTCs as diagnostic biomarkers for patients with advanced late stage cancers. However, CTC detection in early stage disease remains challenging [16] despite the newly-developed technologies in cell isolation and enrichment, and analysis methods (reviewed in [106]). CTC isolation and enrichment techniques include microfluidics-based approaches, surface marker selection of tumor cells, size-based filtration methods such as ISET (isolation by size of epithelial tumor cells) [107], and cell exclusion through negative depletion of immune blood cells using anti-CD45 antibodies [106, 108]. Microfluidics-based platforms utilize magnetic and/or electrophoretic separation systems to capture antibody-tagged CTC complexes [109] but recent developments have enabled isolation based on biophysical characteristics of tumor cells. One example of method development in this area is the slanted spiral microfluidics technique, which has shown high recovery rate (>80%) of CTCs whilst depleting 99.9% of white blood cells from blood [110, 111]. CellSearch (Veridex) is an FDA-approved technique using EpCAM coated beads to isolate CTCs from blood but this surface marker selection is limited to carcinomas that expresses EpCAM [104], and currently, this technique is only approved for prognostic evaluation of metastatic breast, prostate, lung and colon cancer.

Circulating melanoma cells (CMCs) have been detected in blood by qPCR of melanocyte specific genes or by enrichment using melanocyte surface markers; CMCs have shown prognostic value in identifying disease stage [112], progression [113, 114] and overall survival [115] but, sensitivity of CMC detection is low (reviewed in [115, 116]). It remains to be determined whether improvements in enrichment protocols and detection sensitivity could improve CMC detection, especially in early stage melanoma, and currently there has been limited implementation of CMC analysis in the clinic.

#### Circulating immune cells

The presence of tumor-infiltrating immune cells predicts melanoma response to immunotherapy [117–119] and the possibility of using immune cell profiling as a surrogate for tumor tissue analysis is appealing. The cellular complexity of blood requires the use of advanced technologies to detect and discriminate multiple cell populations simultaneously.

The capacity to profile different immune populations has vastly improved due to refinements in multiparameter flow cytometry and mass cytometry techniques. These technologies allow phenotypic and functional characterization of individual cells using multiple parallel tags. Flow cytometry uses antibodies conjugated to fluorochromes that bind to cell surface or intracellular markers to allow cellular characterization; these fluorochromes have now expanded to allow for routine analysis of up to 15 different parameters [120, 121]. Mass cytometry (CYTOF, cytometry by time of flight) also profiles single cells with high throughput and multiple parameters. Instead of using antibodies conjugated to fluorochromes, these antibodies are attached to heavy metal ions that can then be identified using mass spectrometry. Using these metal-conjugated antibodies, mass cytometry has surpassed the multiplexing capacity of flow cytometry, offering up to 40 different parameters [122, 123]. Standardized panels have now been developed for PBMCs and whole blood immunophenotyping [124] and these panels were recently used in the diagnosis of blood-based cancers such as leukemia and lymphoma [125]. There is also potential in combining these platforms to incorporate identification of peptide-MHC multimers in order to characterize reactivity of specific T cell subsets [126, 127].

Several recent studies have demonstrated the utility of immune profiling to discover predictive melanoma biomarkers. In a study with 209 melanoma patients treated with ipilimumab, low absolute monocyte counts, and high absolute eosinophil count, T regulatory cells and relative lymphocyte counts were associated with a favorable outcome [128]. Similarly, in 616 patients treated with pembrolizumab, high relative eosinophil and lymphocyte counts were associated with favorable overall survival [129]. Immune cell phenotyping of PBMCs from patients with stage IV melanoma before and after treatment with anti-PD-1 therapy identified a reinvigorated exhausted CD8+ T cell subset (Eomes^hi^ and Tbet^lo^) expressing the proliferative marker Ki67. Numbers of circulating Ki67+ CD8+ T cells correlated with tumor burden before and after therapy, and more importantly, a ratio of T cell reinvigoration to tumor burden greater than 1.94 significantly associated with better objective response, progression free survival and overall survival [130]. Whether immune profiling of liquid biopsies will be implemented in standard clinical practice will depend on additional studies to validate the predictive value of these immune cell biomarkers. However, it is apparent that an increasing number of clinical trials are including analysis of absolute lymphocyte count as part of their analytical pipeline (Table 1).

## Conclusions

The identification and validation of diagnostic, prognostic and predictive biomarkers are essential for directing and optimizing personalized therapy. For instance, in melanoma, biomarkers that predict and monitor responses to immunotherapies (i.e. ipilimumab, nivolumab and pembrolizumab) will enable the selection of patients most likely to respond to each therapy, identify patients who may require more toxic, combination therapies and ensure efficient use of health care resources; in Australia, pembrolizumab costs over $130,000 per patient per year. These therapies can continue for years, can be given intermittently, or can be used as second-line therapies to prolong patient survival. The management of these long-term cancer survivors requires ongoing review and monitoring, and this can be ideally achieved with inexpensive, accurate and non-invasive liquid biomarkers. Unfortunately, the identification of biomarkers in liquid biopsies has been slow and challenging despite recent advances in molecular and proteomic technologies. Many candidate biomarkers have been identified and proposed but few have reached clinical application. Major factors hindering the approval of new biomarkers include lack of reproducibility, absence of technical standardization, and inadequate validation studies. Given the heterogeneity of individual patients and individual tumors, robust validation of candidate biomarkers requires large-scale prospective multi-center clinical trials. The regulatory pathways involved in biomarker licensing and implementation are also complex and the FDA has published guidelines to support the pre-market development of companion diagnostics.

Liquid biopsy biomarkers could pave the way to better personalized treatment strategies for melanoma patients. Using circulating biomarkers, we may be able to offer patients minimally-invasive, inexpensive and accurate means of selecting the best treatment option and monitoring response during the course of treatment. The identification of potential biomarkers will increase with more sophisticated profiling technologies and more studies focused on the expanding repertoire of targeted and immunotherapies, used alone or in conjunction, in clinical trials. However, biomarker discovery alone is not sufficient, and more emphasis needs to be directed at validation of newly emerging biomarker candidates to realize their implementation into clinic.
